# Impact of Commercialized Genomic Tests on Adjuvant Treatment Decisions in Early Stage Breast Cancer Patients

**DOI:** 10.1155/2020/9238084

**Published:** 2020-11-28

**Authors:** Hazem I. Assi, Ibrahim A. Alameh, Jessica Khoury, Nour Abdul Halim, Fadi El Karak, Fadi Farhat, Juliett Berro, Eman Sbaity, Maya Charafeddine, Arafat Tfayli, Ziad Salem, Nagi El Saghir

**Affiliations:** ^1^Department of Internal Medicine, Division of Hematology and Oncology, Naef K. Basile Cancer Institute, American University of Beirut Medical Center, Beirut, Lebanon; ^2^Department of Internal Medicine, Division of Hematology and Oncology, Hotel Dieu de France University Hospital, Beirut, Lebanon; ^3^Department of Internal Medicine, Division of Hematology and Oncology, Hammoud Hospital University Medical Center, Saida, Lebanon; ^4^Department of Surgery, Division of General Surgery, American University of Beirut Medical Center, Beirut, Lebanon

## Abstract

**Introduction:**

Advances in genomic techniques have been valuable in guiding decisions regarding the treatment of early breast cancer (EBC) patients. These multigene assays include Oncotype DX, Prosigna, and Endopredict. There has generally been a tendency to overtreat or undertreat patients, and having reliable prognostic factors could significantly improve rates of appropriate treatment administration. In this study, we showcase the impact of genomic tests on adjuvant treatment decisions in EBC patients.

**Materials and Methods:**

This is a retrospective study that includes EBC patients treated between December 2016 and February 2018. The physician's choice of treatment was recorded before and after obtaining the results of the genomics tests. Baseline demographics and pathological data were collected from medical records.

**Results:**

A total of 75 patients were included. Fifty patients underwent Oncotype DX genomic analysis, 11 patients underwent Prosigna analysis, and 14 patients underwent Endopredict analysis. A total of 21 physicians' plans (28%) were initially undecided and then carried out after obtaining genomic test results. 13 patients were planned to undergo endocrine therapy alone, while 8 were planned to undergo both endocrine therapy and chemotherapy. Treatment was changed in 26 patients (34.67%). The decision to deescalate therapy was taken in 19 patients (25.33%). The decision to escalate treatment was made in 7 patients (9.33%).

**Conclusion:**

Our study demonstrates the importance of genomics testing, as it assisted physicians in avoiding unnecessary adjuvant chemotherapy in 25.33% of patients, thus reducing side effects of chemotherapy and the financial burden on patients.

## 1. Introduction

Breast cancer is the most common cancer in women worldwide [1]. During the past years, mortality from breast cancer has decreased due to early detection and use of adjuvant systemic therapy. Factors such as hormone receptor expression, human epidermal growth factor receptor 2 (HER2) amplification, tumor size, the involvement of lymph nodes, and histologic grade have been used to determine whether patients with early breast cancer should be treated with chemotherapy, endocrine therapy, or HER2 directed treatments [2]. Recent data that have focused on identifying patients with different recurrence risks after primary surgical therapy using gene-expression profiling have helped clinicians in selecting the optimal treatment for each patient while avoiding overtreatment and reducing toxicities and exposure to chemotherapy [3, 4]. Many patients who have been diagnosed with early-stage breast cancer (EBC) do not derive significant benefit from adjuvant chemotherapy as compared to those receiving endocrine therapy alone. This observation has paved the way for research centered around the development of several genomic aimed at supporting the clinical decision-making process [5]. Subsequently, several genomics tests have been introduced to the market.

Currently, in many countries, the decision to give adjuvant chemotherapy depends on several factors, the most important being the clinicopathological profile. Through this study, we seek to determine the impact that genomics testing has on physicians' treatment decisions at the American University of Beirut Medical Center (AUBMC), and the percentage of patients for whom treatment was deescalated or escalated. We aim to describe the implications of genomics testing on the decision to administer adjuvant treatment in a group of patients in Lebanon, and their potential role in Low-to-Middle Income Countries (LMIC) where data is scarce.

The genomics tests most used in Lebanon include Recurrence Score RS-21 (Oncotype DX), PAM 50 (Prosigna), and Epclin Risk Score (Endopredict). The Oncotype Dx 21-gene recurrence score (RS) assay is the most commonly used genomic assay for ER-positive cancer patients. It utilizes reverse-transcriptase (RT)-PCR to measure the messenger RNA levels of sixteen cancer genes and five housekeeping genes. An algorithm is then used to compute a RS from 0 to 100, which can categorize patients into low-risk (≤17), medium risk (18–30), or high risk (>30) based on the initial National Surgical Adjuvant Breast and Bowel Project (NSABP) ranges [6]. EndoPredict assay is an RNA based assay that measures eight cancer genes in addition to three housekeeping control genes using reverse transcriptase-polymerase chain reaction to calculate EndoPredict score [6]. Patients who have an EP (molecular) score of <5 or Epclin score (molecular and clinical score) <3.3 are considered low risk for late recurrence, while patients who have an EP score >5 or Epclin ≥3.3 are classified as high risk [7, 8]. The Predictor Analysis of Microarray 50 (PAM50), also known as Prosigna kit, is another genomic test that uses an algorithm to compute a risk of recurrence score (ROR) based on quantifying mRNA expression of 50 genes along with intrinsic subtype and tumor size to place patients into high-, intermediate-, and low-risk groups [9]. Patients are classified into low (0–40), intermediate (41–60), or high (60–100) 10-year risk of distant recurrence [10].

So far, the criteria for the use of these tests are somewhat similar and include patients who have early stage cancer (I or II), negative lymph node (LN), or one to three positive LN, hormone receptor (HR) positive and HER2 negative status [9].

## 2. Materials and Methods

The study includes patients diagnosed with EBC at AUBMC and treated between December 2016 and February 2018. The eligibility criteria were the following: women above the age of 18 with EBC (T1-2 N0, including T1pN1mic) that is hormone receptor-positive and HER2-negative. All patients included had undergone a total or partial mastectomy. After assessing pathology, the genomic analysis was carried out on the surgical specimens. We made sure to obtain Institutional Review Board (IRB) approval at our institution before proceeding with patient recruitment. Demographics and clinicopathological data were obtained from a review of charts. Physicians' treatment plans and decisions were recorded as noted before the genomics testing was done and after results were available. The physicians' choice of treatment was divided into three categories: those who planned to give endocrine therapy (ET) only, those who planned to provide endocrine therapy with chemotherapy (ET + CT), and those who were undecided regarding the treatment regimen and wanted to wait for genomic profiling test results. All analyses were performed using IBM SPSS Statistics v.25. Fisher's exact test calculated a statistically significant difference indicated by a *p* value of less than 0.05.

**Figure 1:**
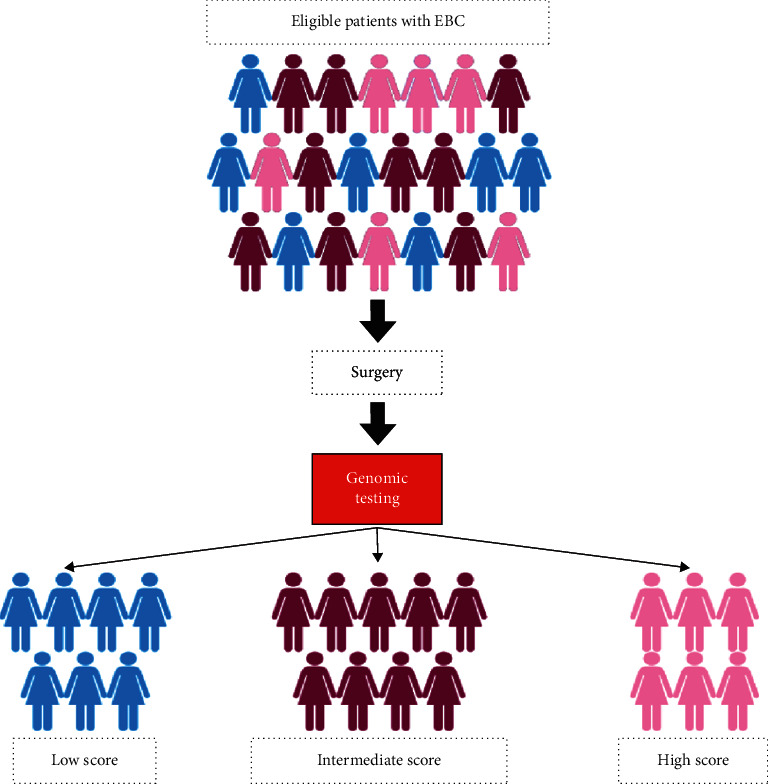
Study process and different score categories.

The genomics tests helped place patients in 3 different categories; low, intermediate, and high recurrence scores (Figure 1).

## 3. Results

A total of 75 patients were included in the study. It was found that the most commonly utilized assay was the Oncotype DX (RS-21) which was used in 50 patients (66.67%), followed by the Endopredict test used in 14 patients (18.67%), and lastly, Prosigna (PAM 50), which was used in 11 patients (14.67%). For the Oncotype DX genomics assay, 21 patients had a low recurrence score, while 26 patients had an intermediate recurrence score, and three patients had a high score. As for the Endopredict assay, ten patients had a low score, and 4 had a high score. As for the Prosigna assay testing, three patients had low scores, while seven patients had intermediate scores, and only one patient had a high score.

The mean age of the population was 51.67 years, and the age range of patients was between 31–81 years. Most patients (84.93%) had low-grade tumors (grades 1 and 2), while 15.06% had high grade tumors (grade 3) (Table 1).

According to our results, ten patients were planned to receive endocrine therapy alone before they underwent genomics analysis, and after the genomics analysis, 3 of the physicians did not change their plans and proceeded with giving endocrine therapy only, while the other seven decided to change their patients from taking only endocrine therapy to taking both endocrine therapy and chemotherapy.

Out of these 44 patients who were planned to receive chemotherapy and endocrine therapy prior to genomic analysis, 19 patients underwent treatment deescalation and were given endocrine therapy only while the other 25 patients received the initial treatment plan.

A total of 21 physicians' plans (28%) were undecided. After the genomics tests were carried out, 13 patients were planned to undergo endocrine therapy alone, while eight were scheduled to undergo both endocrine therapy and chemotherapy.

**Figure 2:**
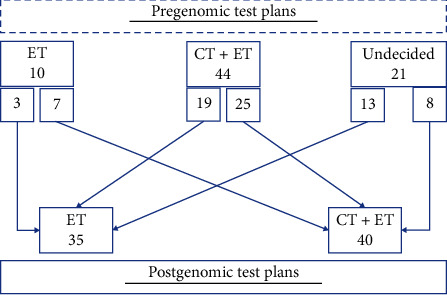
Pregenomic and postgenomic treatment plans. ET = Endocrine Therapy; CT = Chemotherapy.

Treatment was changed in 26 patients (34.67%). The decision to deescalate therapy was taken in 19 patients (25.33%). The decision to escalate treatment was made in 7 patients (9.33%). The use of genomic assays helped in deciding treatment for 21 patients (28%) (Figure 2).

All patients were split into three categories: low, intermediate, and high-risk groups based on the ranges of each genomics assay.

As shown in Table 2, treatment deescalation in the low score category occurred in 12 patients out of the 19. All undecided cases [10] were given endocrine therapy after undergoing genomic tests. A total of 7 patients in the low score category ended up receiving chemotherapy.

In patients lying in the intermediate-risk category, treatment was escalated in the four patients who were planned to receive ET alone prior to testing. Moreover, 6 out of 10 patients in the undecided group were given ET + CT following genomic testing. Deescalation occurred in 4 out of 19 patients in the ET + CT pregenomic plan category.

A total of 8 patients ended up receiving ET after the genomics assay test, and 25 patients received ET + CT.

As for patients in the high-risk category, treatment decisions were kept the same for the seven patients before and after the genomic assay test. Moreover, genomic testing helped in deciding treatment for one patient who ended up receiving ET + CT.

According to originally proposed RS cut-offs, 17 of 21 patients who were classified as low RS received endocrine therapy alone, while four received chemotherapy in addition to endocrine therapy. Moreover, 19 patients in the intermediate RS category received a combination of endocrine and chemotherapy. All the patients in the high RS category received chemotherapy (Table 3).

**Figure 3:**
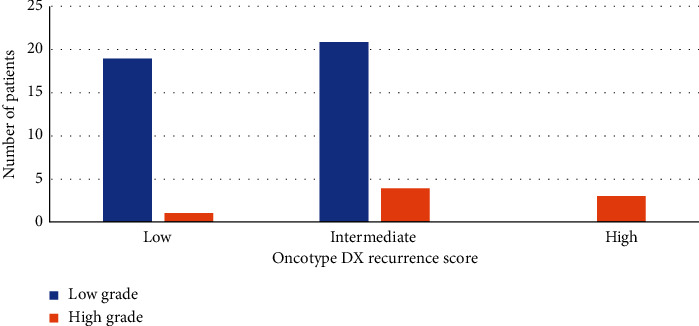
Oncotype DX RS in relation to tumor grade.

We examined the correlation between the Oncotype DX RS and tumor grade, as seen in Figure 3. Most patients who had high-grade tumors were found to have an intermediate or high RS (*p* value <0.001). Only one patient with a high-grade tumor was classified in the Low RS category. Likewise, patients who had low-grade tumors had a low or intermediate RS following genomic testing. The RS was dependent on the histologic grade.

**Figure 4:**
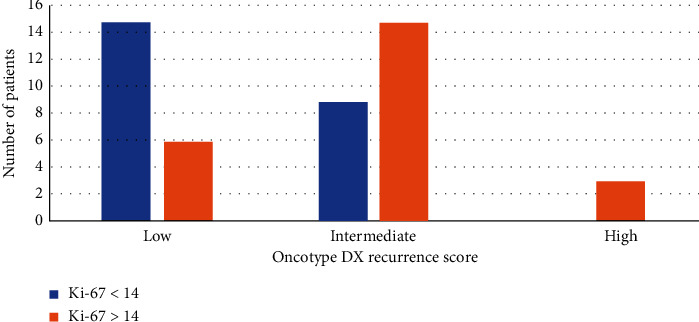
Oncotype DX RS in relation to Ki-67.

In addition to that, we examined the correlation between the Oncotype DX RS and Ki-67. As seen in Figure 4, Ki-67 was significantly associated with RS categories (*p* value <0.05). All patients who had Ki-67 less than 14 ended up having a low or intermediate RS. Also, all patients with high RS had Ki-67 > 14.

## 4. Discussion

The decision to administer chemotherapy to a cancer patient and the decision to avoid it is very challenging. Breast cancer patients frequently come to their first appointment with prior knowledge regarding their disease and the risks and benefits of chemotherapy. Some patients hope to avoid chemotherapy at all costs fearing its side effects. In contrast, others fear cancer recurrence and death and opt to pursue all available treatment regardless of the associated toxicities. Genomic test results can help improve the patient's understanding of her recurrence risk and the benefit of chemotherapy, with the goal being to improve treatment decision-making.

In our population, genomic testing helped make a treatment decision or change the therapeutic plan in 62.67% of patients (*n* = 26 and *n* = 21, respectively). As seen in Figure 2, nineteen patients (25.33%) underwent deescalation of treatment and avoided chemotherapy. This percentage reflects the importance of genomics testing in tailoring the therapeutic plan for patients with ER-positive and HER2-negative EBC.

We compared our Oncotype Dx findings to another study done by Poorvu et al. [11]. In women who had genomic testing performed, chemotherapy usage was 24%, 57%, and 100% among those with low, intermediate, and high RS, respectively compared to 19%, 73%, and 100% in low, intermediate, and high RS category in our study. As a result, chemotherapy overtreatment continues to occur, as shown in low and intermediate RS categories. It is a reality that should concern all clinicians involved in breast cancer workup and subsequent management. Besides, we observed that seven patients with low score received chemotherapy (Table 2). The reason for having such a significant number receiving chemotherapy was attributed to a borderline low score in some patients, high Ki-67 in young patients, and the presence of micrometastasis in a lymph node. Applying the original Oncotype Dx cut-offs, four patients of the low score group received chemotherapy in addition to endocrine therapy (Table 3). To go further in each patient's case, three patients had microscopic metastasis to lymph nodes and had RS of 8, 14, and 16, respectively. In contrast, the fourth patient was young (41 years old), had a RS of 11, but she preferred to receive chemotherapy, believing that it will reduce the risk of cancer recurrence.

A lot of uncertainty still roamed around treatment plans for patients in the intermediate category, with some physicians preferring to treat with both chemotherapy and endocrine therapy. As seen in Table 3 [12], patients in the intermediate RS category received the combination of chemotherapy and endocrine therapy. In a study that describes the physicians' perspectives when it comes to using genomics tests, respondents noted that the most significant clinical challenge associated with Oncotype DX® was the uncertainty associated with the intermediate RS [13]. After the implementation of TAILORx which was designed to help determine whether chemotherapy is useful for women with RS in the intermediate category, there was less uncertainty about the best treatment for this group, as TAILORx was a prospective randomized clinical trial and a new mid-range intermediate score of 11–25 was used. Women in the trial who had a score of 11 to 25 were randomly assigned to receive endocrine therapy alone or endocrine treatment with adjuvant chemotherapy. It has been shown that endocrine therapy alone, and chemotherapy along with endocrine therapy, had similar efficacy in women with hormone receptor-positive, HER2-negative, axillary node-negative breast cancer in the intermediate range of the 21-gene recurrence score in the overall population. However, a subanalysis showed that a small benefit of 1.6% was noted for patients below 50 with RS-21 between 16-20, and a benefit of 6.5% was noted for patients with RS-21 between 21–25 [14]. It is also thought that a large part of the benefit in this intermediate group is potentially due to chemotherapy-induced ovarian function suppression and that more patients may be spared chemotherapy using LHRH antagonists [15]. Therefore, it can be concluded that there is no benefit from receiving chemotherapy plus endocrine therapy for the majority of patients in this intermediate-risk group, as the proportion of women who developed recurrence or a second primary cancer was very similar in both groups [16].

Furthermore, patients who ended up having a high score were the lowest in number (*n* = 8). It can be inferred from this observation that a minimal number of physicians ordered a genomic assay test for patients who ended up being in the high score category. In addition, most patients in the high-risk group were scheduled to undergo chemotherapy before genomic testing (Table 2). It can be assumed from this subgroup that the clinicopathological data is sometimes enough for the physician to choose to give both endocrine therapy and chemotherapy. Presently, additional data is available from the MINDACT study that shows that 46% of patients with High Clinical Risk turn out to be of Low Genomic Risk using the Amsterdam-70 gene (Mammaprint) genomic profiling test and may be spared chemotherapy [17].

Histological findings such as mitosis, nuclear atypia, and tubule formation that are indirectly related to individual genes (hormone receptors and proliferation) are computed in the measuring of histological grade. The same findings are the products contributing to Oncotype Dx [18, 19]. One of the most significant predictors of RS is the histologic tumor grade [12, 18]. Also, the 21-gene panel of the Oncotype Dx assay includes the Ki-67 gene, but its gene expression is reported as part of the RS and not individually [20]. Thus, a correlation must be found between RS on one hand and Ki-67 and tumor grade on the other. As shown in Figures 2 and 3, all the patients in the high RS categories had high-grade tumors and Ki-67 greater than 14. This could be useful in some LMIC where the government does not cover genomics testing, and the cost of these tests forms an additional burden on the patient. In cases of high-grade tumors with Ki-67 > 14, patients benefit from chemotherapy, as the RS will most probably be high. Other studies have shown that very low or very high Ki-67 correlates well with low or high-risk RS-21 [21]. Chemotherapy regimens for breast cancer are based on anthracyclines and taxanes, but the main problem was the lack of biomarkers predicting the efficacy of these drugs. Some observations were made that anthracycline-based regimens had increased efficacy in patients with HER2 overexpression as it could be caused by the amplification of the TOP2A gene which has been found to be amplified in 35% of these cases [22–25]. Genomic profiling is still not valid to select specific chemotherapy regimens during the clinical care of patients with breast cancer. Moreover, gene signatures based on DNA damage repair pathways have been reported as potential biomarkers of anthracyclines in ER positive and negative breast cancer [26]. On the other hand, some hypothesis proclaimed that taxanes might have optimal efficacy in chromosomally stable, low-grade tumors. These hypotheses were supported by the Early Breast Cancer Trialists' Collaborative Group (EBCTCG) evidence that taxane-based therapy offers the greatest relative risk reduction to women with low-grade ER-positive breast cancer. Furthermore, lower assay scores might reflect relative taxanes benefit. For the PAM50 assay, phase III GEICAM/9906 trial showed the benefit of paclitaxel in low PAM50 score [27]. Based on this discussion, there is a need for explicit trials tailoring the use of the optimal chemotherapy regimen, depending on the attributed genomic score.

Currently, the American Society of Clinical Oncology (ASCO) strongly recommends the use of genomic tests to help guide adjuvant chemotherapy decisions in ER-positive, HER2-negative, node-negative breast cancer. Also, the National Comprehensive Cancer Network (NCCN) recognizes it as the only genomic assay proven to predict chemotherapy benefit, incorporating it into its guidelines for use in patients with ER-positive, HER2-negative tumors greater than 0.5 cm and pathologic node-negative or micrometastatic nodal disease [1, 11].

## 5. Conclusion

When it comes to treatment decisions, traditional clinical and pathological variables remain standard references. In addition to that, the use of multigene assays to assess the intrinsic molecular characteristics of ER-positive, HER2-negative tumors in early-stage breast cancer patients has led to a more precise risk stratification for recurrence at the individual patient level.

Our study demonstrates the real-world importance of genomic profiling testing as it assisted treatment decisions for 21 patients (28% of total) in whom physicians were undecided, and it helped avoid unnecessary adjuvant chemotherapy in 25.33% of patients in whom physicians were planning to give it. It also played a crucial role in identifying patients who are likely to benefit from chemotherapy, and for whom chemotherapy is unlikely to be offered if the physician were to rely on clinicopathological diagnosis alone (9.33%). Therefore, the genomics tests will likely be a central asset in preventing overtreatment and undertreatment. We emphasize the importance of taking a global view that incorporates the recurrence score in addition to the clinical and histological factors, patient's choice, performance status, and comorbidities when deciding for treatment [28]. Our study shows the impact of the genomic assay score results on treatment decisions and physicians' choices, and the need to discuss treatment options with well-informed patients as part of the physician-patient treatment decision-making process.

## Figures and Tables

**Table 1:** Baseline characteristics of the patient.

Characteristics	Patients
Age (years), mean	51.67 (31–81)
*Primary surgery*, *n(%)*	
Total mastectomy	25 (33.33)
Partial mastectomy	50 (67.67)
*Histology, n (%)*	
Invasive ductal carcinoma	63 (84.00)
Invasive lobular carcinoma	12 (16.00)
*Presence of DCIS, n (%)*	
No	30 (40.00)
Yes	45 (60.00)
*Tumor grade, n (%)*	
Low grade	62 (84.93)
High grade	11 (15.06)
*Tumor size, n (%)*	
<2 cm	44 (59.46)
2–5 cm	30 (40.54)
*Ki-67*	
<14	35 (49.30)
>14	36 (50.70)
*Oncotype, n*	
<18	21
18–31	26
>31	3
*Endopredict, n*	
Low	10
High	4
*Prosigna, n*	
Low	3
Intermediate	7
High	1

**Table 2:** Therapeutic plans pregenomics and postgenomics testing according to scores for 75 patients.

		Undecided	ET	ET + CT
Low score	Pregenomic plan	10	5	19
	Postgenomic plan	0	27	7
Intermediate score	Pregenomic plan	10	4	19
	Postgenomic plan	0	8	25
High score	Pregenomic plan	1	0	7
	Postgenomic plan	0	0	8

**Table 3:** Therapeutic plans pregenomics and postgenomics testing according to Oncotype DX Recurrence Scores for 50 patients.

		Undecided	ET	ET + CT
Low RS	Pregenomic plan	6	3	12
	Postgenomic plan	0	17	4
Intermediate RS	Pregenomic plan	7	3	16
	Postgenomic plan	0	7	19
High RS	Pregenomic plan	1	0	2
	Postgenomic plan	0	0	3

## Data Availability

The data used to support the findings of this study are available from the corresponding authors upon request.
